# Oxygen production from dissociation of Europa’s water-ice surface

**DOI:** 10.1038/s41550-024-02206-x

**Published:** 2024-03-04

**Authors:** J. R. Szalay, F. Allegrini, R. W. Ebert, F. Bagenal, S. J. Bolton, S. Fatemi, D. J. McComas, A. Pontoni, J. Saur, H. T. Smith, D. F. Strobel, S. D. Vance, A. Vorburger, R. J. Wilson

**Affiliations:** 1 Department of Astrophysical Sciences, Princeton University, Princeton, NJ USA; 2 Southwest Research Institute, San Antonio, TX USA; 3 Department of Physics and Astronomy, University of Texas at San Antonio, San Antonio, TX USA; 4 Laboratory for Atmospheric and Space Physics, University of Colorado Boulder, Boulder, CO USA; 5 Department of Physics, University of Umeå, Umeå, Sweden; 6 Institute of Geophysics and Meteorology, University of Cologne, Cologne, Germany; 7 The Johns Hopkins University Applied Physics Laboratory, Baltimore, MD USA; 8 The Johns Hopkins University, Baltimore, MD USA; 9 Jet Propulsion Laboratory, California Institute of Technology, Pasadena, CA USA; 10 Physics Institute, University of Bern, Bern, Switzerland

**Keywords:** Rings and moons, Magnetospheric physics

## Abstract

Jupiter’s moon Europa has a predominantly water-ice surface that is modified by exposure to its space environment. Charged particles break molecular bonds in surface ice, thus dissociating the water to ultimately produce H_2_ and O_2_, which provides a potential oxygenation mechanism for Europa’s subsurface ocean. These species are understood to form Europa’s primary atmospheric constituents. Although remote observations provide important global constraints on Europa’s atmosphere, the molecular O_2_ abundance has been inferred from atomic O emissions. Europa’s atmospheric composition had never been directly sampled and model-derived oxygen production estimates ranged over several orders of magnitude. Here, we report direct observations of H_2_^+^ and O_2_^+^ pickup ions from the dissociation of Europa’s water-ice surface and confirm these species are primary atmospheric constituents. In contrast to expectations, we find the H_2_ neutral atmosphere is dominated by a non-thermal, escaping population. We find 12 ± 6 kg s^−1^ (2.2 ± 1.2 × 10^26^ s^−1^) O_2_ are produced within Europa’s surface, less than previously thought, with a narrower range to support habitability in Europa’s ocean. This process is found to be Europa’s dominant exogenic surface erosion mechanism over meteoroid bombardment.

## Abstract (web summary)

Water molecules in Europa’s icy surface are split into hydrogen and oxygen by charged particle bombardment. NASA’s Juno spacecraft flew near Europa and constrained the production of oxygen in Europa’s surface ice, thus providing only a narrow range to support habitability in its subsurface ocean.

## Main

Europa’s interaction with its space environment, notably charged particles, ultraviolet light and meteoroid impacts, modifies its surface chemistry, leading to erosion and deposition of exogenic material. Charged particles dissociate H_2_O in the surface ice (breaking molecular bonds), which subsequently recombine predominantly into molecular H_2_ and O_2_ (refs. ^1,2^). These molecular species are expected to be dominantly released from the surface by thermal desorption^2–4^. Thermal desorption along with sputtering from electrons^5^ or ions^6,7^ can liberate these molecules from the surface into Europa’s atmosphere. This atmosphere is understood to comprise H (ref. ^8^) and H_2_, O and O_2_ (refs. ^9–12^), and H_2_O (refs. ^12–14^). Atmospheric neutrals can become ionized as pickup ions (PUIs) that are incorporated into Jupiter’s magnetospheric plasma^15–18^. Atmospheric sputtering, in which a plasma exchanges momentum with and erodes the neutral atmosphere, was originally proposed to be the dominant loss mechanism of neutral O_2_^15^. Subsequently, electron impact ionization^3^ and symmetric O_2_^+^ → O_2_ charge exchange^19^ have also been proposed as the primary drivers of O_2_ loss. H_2_ loss has been less investigated theoretically; however, electron impact ionization is proposed to be its dominant loss mechanism^3^.

The atmosphere is understood to consist of thermally desorbed molecules. It is governed by the surface temperature^20^ as well as a directly sputtered source^21^. Although Galileo’s E4 and E6 fly-bys at close-approach altitudes of 692 and 586 km inferred the presence of PUIs near Europa, instrumental limitations prevented a compositional deconvolution of the measured plasma into magnetospheric and Europa-genic material^22^. Additionally, several species of PUIs were inferred from ion-cyclotron emissions during the E11 and E15 fly-bys^23^. Constraints on the relative abundances of Europa’s atmospheric neutral and plasma species were previously derived primarily from remote-sensing ultraviolet observations. As there had been no direct in situ particle observations of Europa-genic material composition in the moon’s vicinity, the composition of Europa’s atmosphere, how much of it is lost and how much plasma Europa contributes to Jupiter’s magnetosphere remained unresolved^24^.

## Observations and PUI characteristics

**Fig. 1:**
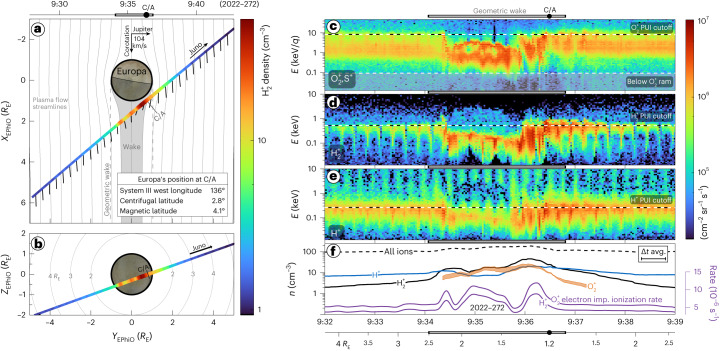
Overview of Europa fly-by and plasma observations. **a**,**b**, Density of H_2_^+^ PUIs directly picked up from Europa’s neutral atmosphere for *X*_EPhiO_ (**a**) and *Z*_EPhiO_ (**b**). Velocity arrows indicate the plasma velocity vector as determined from proton observations, with the rigid corotation of 104 km s^−1^. *R*_E_ ≡ 1,560.8 km is Europa’s radius. Streamlines and associated wake are from an analytic model (Methods). **c**–**e**, Fluxes of O_2_^+^ and S^+^ (**c**), H_2_^+^ (**d**) and H^+^ (**e**) from JADE’s TOF product. Horizontal dashed lines indicate the ram energy for O_2_^+^ (**c**) and cutoff energies (**c**–**e**) for PUIs assuming rigid corotation (Methods). **f**, Densities of individual species (orange, black and blue), all ions (dashed black) and electron impact ionization rates (purple, right axis). The altitude is shown underneath **c**–**f**. The boundaries of the geometric wake are shown with horizontal grey bars above or below each panel. Juno’s close approach (C/A) was on 2022-272 9:36:29. Each horizontal tick corresponds to 1 min. avg., average; imp., impact. Source data

The Juno mission^25^ is equipped with the Jovian Auroral Distributions Experiment (JADE)^26^, which includes several electron analysers and a time-of-flight (TOF) ion mass spectrometer. JADE’s ion instrument measures the energy and angle distributions of positively charged particles with an energy per charge (*E*/*q*) of 10 to 46 keV/*q*. Juno performed a fly-by of Europa on 29 September 2022 (day of year 272), with its closest approach at 9:36:29 UTC at an altitude of 353 km and a radial distance of 1.2 *R*_E_, where 1 *R*_E_ = 1,560.8 km. Relevant orbital parameters are given in Extended Data Table 1. Figure 1a,b shows the fly-by trajectory in Europa phi orbital (EPhiO) coordinates, where +*z* is aligned with Jupiter’s rotation axis, +*y* is the direction of the component of the Europa–Jupiter vector perpendicular to +*z* and +*x* completes the right-handed system, which is aligned with the rigid corotation direction.

Juno transits the geometric wake from 9:34:06 to 9:36:48 UTC with a speed relative to Europa of 23.6 km s^−1^. Figure 1c–f shows plasma observations from JADE. The fluxes are derived by integrating the TOF data to identify H^+^, H_2_^+^, and O_2_^+^/S^+^ (Methods) to focus on Europa-genic PUI species. The upstream densities of ~100 cm^−3^ at Europa during this fly-by are within the 25–50% range of densities observed over Galileo’s tour^27^. This plasma density contrasts with Galileo’s E4 fly-by, which had a similar fly-by geometry, but with much lower upstream total ion densities of ~20 cm^−3^ (ref. ^22^). It also contrasts with Galileo’s E12 fly-by when Europa was near the plasma sheet. Its plasma waves spectrometer observed large densities >600 cm^−3^ before the transit, and <200 cm^−3^ after. However, this enhancement may have been due to activity at Europa^28^, and the E12 density profile is markedly different than that observed during Juno’s Europa fly-by.

The expected PUI cutoffs (Methods) for rigid corotation of 104 km s^−1^ at Juno are 0.3, 0.5 and 8 keV for H^+^, H_2_^+^ and O_2_^+^, respectively, as shown in the horizontal dashed lines in Fig. 1c–e, which also shows the ram energy for O_2_^+^ of 90 eV. Ram energies for the hydrogen species are below JADE’s 10 eV/*q* lower limit for ions. Most notably for H_2_^+^ and O_2_^+^, the cutoff in fluxes at the higher-energy range matches almost identically to the expected PUI cutoff (for rigid corotation) outside the Europa transit. Just after crossing into the wake, Juno transits a region with a varying PUI cutoff energy, indicating these ions were picked up at speeds differing from rigid corotation. This corresponds to the speed increasing around the flanks of Europa and the slowing and deflection of plasma within its wake.

**Fig. 2:**
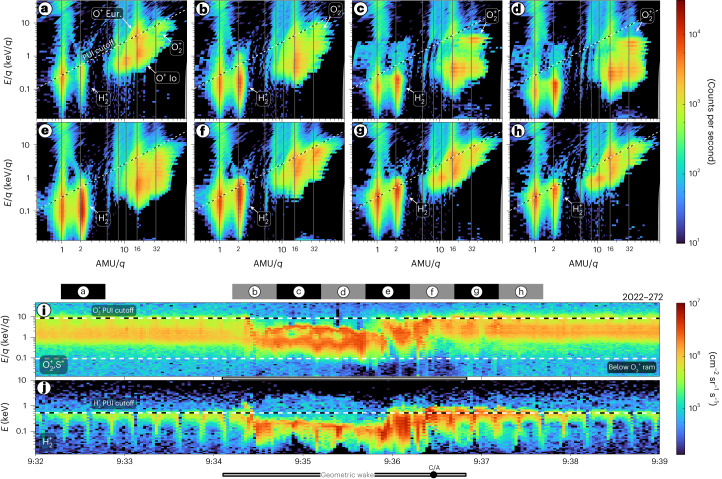
Ion composition for eight periods along the fly-by. **a**–**h**, Average ion count rates as a function of energy per charge and mass per charge. The diagonal line on each shows the cutoff for locally picked up ions assuming that they are picked up at a rigid corotation speed of 104 km s^−1^ relative to Europa (Methods). The corresponding 30 s intervals are indicated on the top of **i**. **i**,**j** The same as shown in Figs. 1c (**i**) and 1e (**j**). Source data

Several species from distinct plasma populations have been observed near Europa. JADE can discriminate these with its TOF observations (Fig. 2). Magnetospheric H^+^, O^2+^, S^3+^, O^+^/S^2+^ and S^+^ are observed consistently throughout the encounter above a few kilo-electronvolts, with a depletion below these energies within Europa’s wake. Notably, S^3+^ at *M*/*q* = 10.67 (atomic mass unit per elementary charge) is an important tracer for magnetospheric plasma as there is no appreciable source of sulfur from Europa compared to the Io-genic plasma dominating the magnetosphere. In contrast, H_2_^+^ of Europa-genic origin is more prominently observed closer to Europa where the magnetospheric plasma populations are depleted. For O^+^, both Europa-genic ions near the PUI cutoff energy and Io-genic O^+^ near the corotation speed are observed. For example, Fig. 2a shows both O^+^ populations separated by energy. The expected Europa plasma torus H_2_^+^ densities at Europa’s orbit are 0.2–0.3 cm^−3^ (ref. ^29^), which are negligible compared to the H_2_^+^ densities of ~2–60 cm^−3^ observed here.

**Extended Data Fig. 1:**
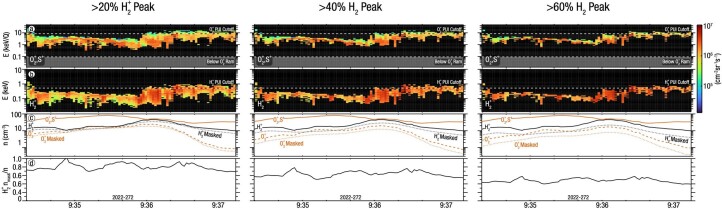
Masking analysis for O_2_^+^ density estimation. Panels (a) and (b) show identical data in panels (a) and (c) in Fig. 1, where a mask has been applied to each determined by the percentage from peak flux in H_2_^+^. The time range shown is 2022-272 9:34:20 to 9:37:15, where modified M/q = 32 fluxes were observed above the background magnetospheric heavy ions. Panel (c) shows the densities derived solely from the masked data (dotted lines) and those corrected for the missing portion of the distribution (dashed lines) using the correction factor shown in panel (d) from H_2_^+^.

Near Europa there are two distinct ion populations in the *M*/*q* = 32 fluxes that separate in energy. We attribute the lower-energy population to magnetospheric ions and the higher-energy population to O_2_^+^ PUIs. Before and after the encounter, there is a quasi-steady-state population peaking in differential energy flux between 1 and 10 keV. These fluxes probably contain a mixture of both magnetospheric S^+^ ions originating from Io as well as Europa-genic O_2_^+^ PUIs near the PUI cutoff energy observed inside and outside Europa’s orbit. They may also contain a lower level of false coincidences from O^+^ and S^++^ with longer TOFs on the lower end of this energy range. Within the wake, an enhancement is observed in *M*/*q* = 32 fluxes. The magnetospheric O^*n*+^ and S^*n*+^ are all slowed within the wake and observed with lower energies. The higher-energy population in the wake, notably ~2–3 keV/*q* with *M*/*q* = 32, follows very closely to the H_2_^+^ PUI population. If both species were picked up in identical locations and transport conditions, the O_2_^+^ PUIs would have a similar energy distribution upscaled by a factor of 16 in energy for the difference in mass between O_2_ and H_2_, with the exception of additional gyrotropic effects discussed below. As the *M*/*q* = 2 fluxes are unambiguously H_2_^+^ PUIs from Europa, we can use their temporal and energy distribution to constrain Europa-genic O_2_^+^ (Extended Data Fig. 1). Figure 1f shows the range of derived O_2_^+^ densities during the period when the *M*/*q* = 32 energy-per-charge spectrogram (Fig. 1c) is distinct from the upstream conditions, specifically from 2022-272 9:34:20 to 9:37:15. As densities derived for the full *M*/*q* = 32 product would contain contributions from several species, we isolate and show only the O_2_^+^ densities we can derive in this data-driven way. Unlike at Ganymede, where H_3_^+^ was observed^30^, probably being a direct by-product of a relatively dense H_2_ atmosphere, no appreciable signatures of H_3_^+^ were observed during this Europa transit. These observations also provide in situ constraints on PUI currents, the subtraction of which is necessary to better constrain the induced current due to Europa’s subsurface ocean^31^.

Some of the differences between the H_2_^+^ and O_2_^+^ energy distributions may be due to gyrotropic effects^32^. For an average upstream magnetic field magnitude of ~440 nT using the JRM09 internal field model^33^ and current sheet model^34^ and assuming pickup at a rigid corotation of 104 km s^−1^, the gyroradius for H_2_^+^ is ~5 km whereas the O_2_^+^ gyroradius is ~80 km. With a speed relative to Europa of 23.6 km s^−1^, Juno transits a full O_2_^+^ gyroradius every 3–4 s, or two ion measurement periods at 2 s each such that JADE may not be sampling a fully gyrotropic population at any given period, particularly near a close approach where these ions would be the most freshly picked up.

Within the geometric wake, the dominant Europa-genic species are H_2_^+^ and O_2_^+^, with both densities peaking ~30 s before close approach. This confirms that the primary atmospheric neutral constituents are H_2_ and O_2_. With the exception of when Juno was most central to the wake at ~9:35, the O_2_^+^ densities are lower than those observed for H_2_^+^. However, Juno may have missed the densest core of O_2_^+^ in Europa’s wake^16^ due to its fly-by trajectory and three-dimensional nature of the streamlines carrying PUIs.

Juno encountered a diverse and mixed plasma environment with Europa-genic PUIs and magnetospheric plasma at all altitudes visited. The relative ratios of the various constituents vary substantially, such that this convection-driven ionosphere is compositionally stratified. Thus, a meaningful scale height cannot be derived from a single electron density observation^35^. This finding also has important implications for upcoming Europa Clipper and Jupiter Icy moons Explorer (JUICE) fly-bys. Specifically, the energy-per-charge observations with Clipper’s Faraday cup^36^ will need to be carefully interpreted given the overlap of O_2_^+^ PUIs with magnetospheric S^+^.

## Atmospheric properties

**Fig. 3:**
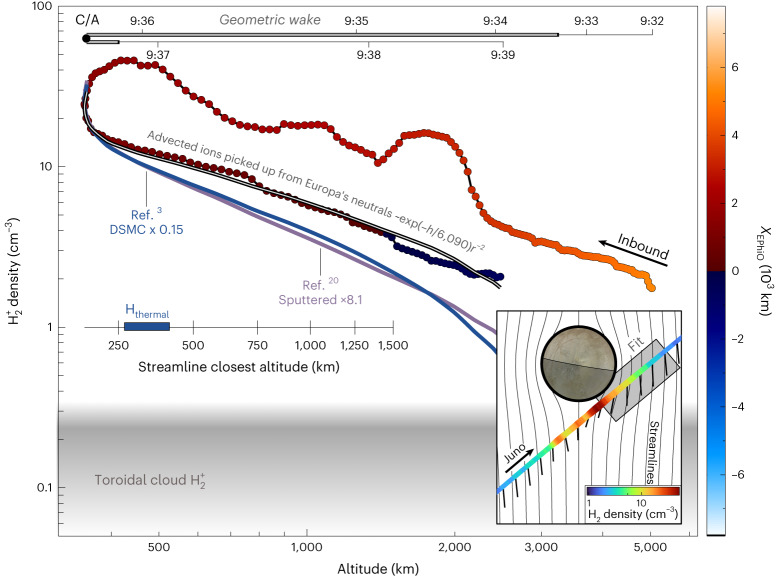
Altitude profile for H_2_^+^ PUIs. H_2_^+^ density coloured by *X*_EPhiO_ such that densities observed upstream from the centre of Europa are blue and are red or orange downstream. The outbound portion of the trajectory, ‘Fit’ in the inset, is compared to a PUI advection solution (Methods). Overlaid curves show PUI densities corresponding to the advection solution for a PUI population from: (1) an ionized neutral atmosphere varying as exp(−*h/λ*)*r*^−*2*^ where *λ* = 6,090 ± 890 km (grey), (2) scaled from a comprehensive DSMC atmosphere model (blue)^3^ and (3) scaled from a sputtered-only model (purple)^20^. The grey region at the bottom shows the expected density of Europa-genic H_2_^+^ PUIs already incorporated into Jupiter’s magnetospheric plasma^29^. Source data

**Extended Data Fig. 2:**
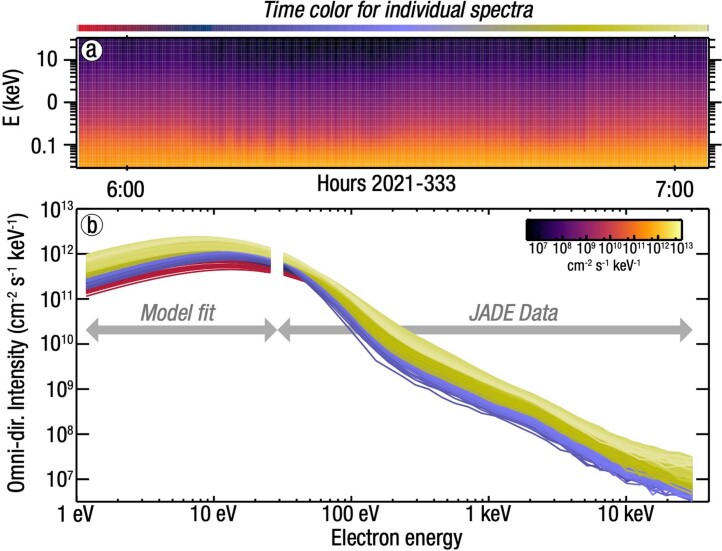
Electron intensity distribution for electron reaction rates. Panel (a) shows example electron spectra when Juno was between r = 9-10 R_J_ and within z = 2 R_J_ from the magnetic equator during the 38^th^ perijove. Panel (b) shows these spectra along with the extrapolated lower energy component of the distribution function by fitting each individual spectrum to a kappa distribution. These extrapolations are used to compute the full electron reaction rate cross-sections.

**Extended Data Fig. 3:**
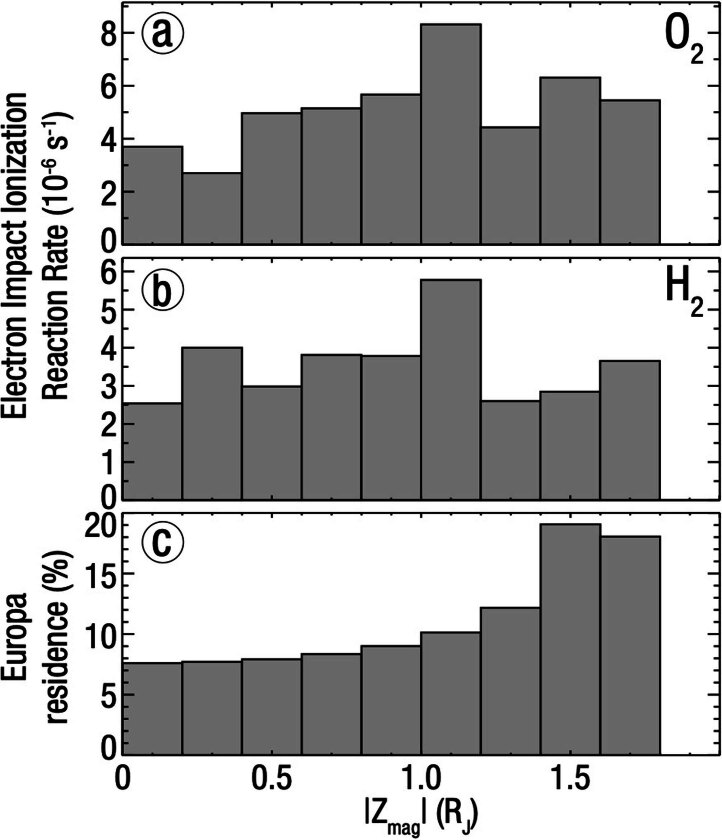
Electron impact ionization rates for H_2_ and O_2_ as a function of z_mag_. Panels (a) and (b) show the average electron impact ionization rates for each separate species derived from Juno/JADE electron measurements in the vicinity of Europa’s orbit (r = 9-10 R_J_ and within z = 2 R_J_). Panel (c) shows the relative residence time Europa spends in each location. These distributions are used to determine the normalized rates in Extended Data Fig. 4.

**Extended Data Fig. 4:**
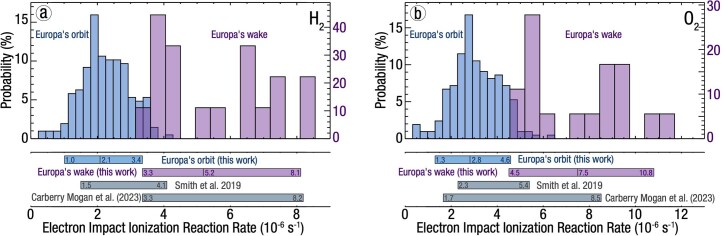
Electron impact ionization rates for H_2_ and O_2_. Panels (a) and (b) show the same information for separate species. The blue histogram shows the probability distribution of total electron impact ionization rates derived from Juno/JADE electron measurements in the vicinity of Europa’s orbit (r = 9-10 R_J_ and within Z_mag_ ≤ 2 R_J_) normalized by time Europa spends as a function of magnetic latitude. The purple histogram shows these values derived when Juno was within Europa’s geometric wake. Widths of the blue and purple bars at the bottom indicate the 10% and 90% percentiles with the median value shown in the central vertical line. Previous estimates in the vicinity of Europa’s orbit are shown in grey^40,63^.

**Extended Data Fig. 5:**
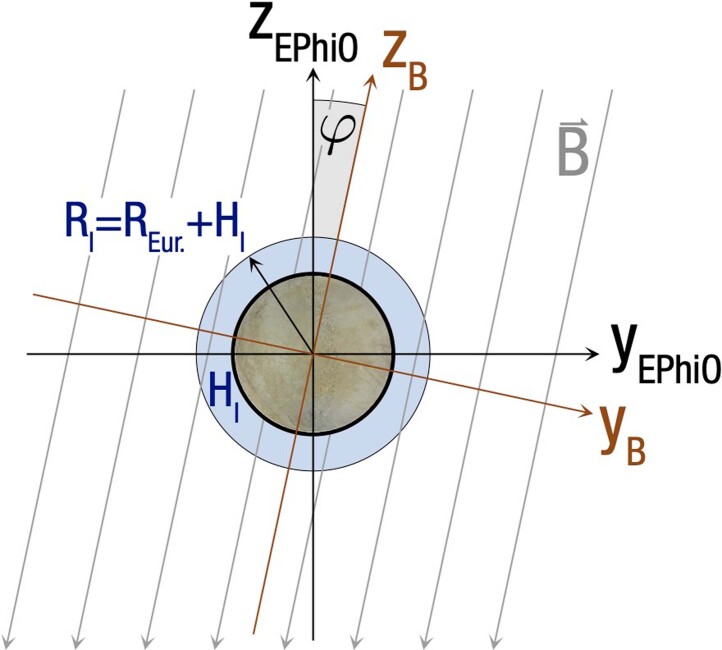
Coordinate systems used to calculate streamlines. Orange axes show the coordinate system where -*z*_B_ is aligned with the magnetic field direction and the component of B out of the page is considered negligible. This coordinate system differs from EPhiO by a rotation by angle $\varphi$ about the *x* direction. The ionospheric altitude *H*_*I*_ and radius *R*_*I*_ are also indicated.

**Extended Data Fig. 6:**
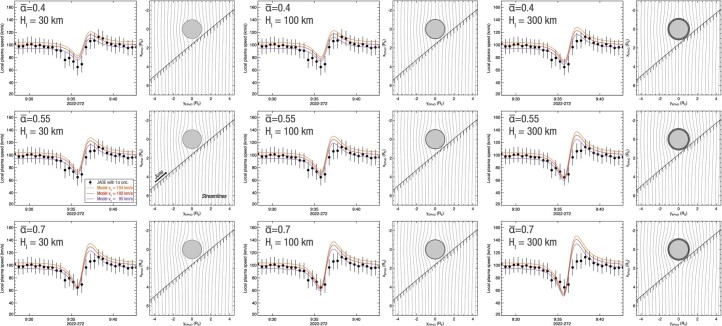
Comparison of JADE plasma flow speeds with streamline model. Each time series panel shows the Juno/JADE derived mean local speed using JADE proton measurements along with the 1σ uncertainties summed in quadrature from values within the JADE data files. The three curves show model predictions for this speed for corotation speeds of 104 km s^−1^ (orange, rigid corotation), 100 km s^−1^ (red), and 95 km s^−1^ (purple). The panel to the right of each time series shows the corresponding streamline model along with Juno’s trajectory and observed velocity vectors. The light grey circle corresponds to Europa and the dark grey annulus corresponds to the modeled ionospheric height H_I_. Nine different model cases are shown for interaction strength *α* = (0.4,0.55,0.7) and *H*_*I*_ = (30 km, 100 km, 300 km). We use values of *α* = 0.55 and *H*_*I*_ = 30 throughout the analysis (middle row, left column).

The observed PUIs can be used to infer atmospheric neutral densities. To do so, we focus on times when Juno was on the Jupiter-facing side of the geometric wake (Fig. 3 inset). This location is where Juno transits streamlines that have the nearest access to the densest portions of the neutral atmosphere, which reduces additional effects due to the complex wake dynamics and enables us to estimate total atmospheric neutral densities upstream along streamlines connected to Juno using a small number of realistic assumptions (Methods). We calculate the electron impact ionization rates (Methods and Extended Data Fig. 2) for all JADE data near Europa’s orbit (Extended Data Table 2 and Extended Data Fig. 3) and during the fly-by (Extended Data Fig. 4), finding this mechanism to be the dominant ionizing process for these neutrals at Europa (Extended Data Table 3), as shown in Fig. 1f. From these rates, we compare modelled PUI densities from an advection model (Methods and Extended Data Figs. 5 and 6) for three specific atmospheric neutral profiles to the H_2_^+^ densities in Fig. 3: (1) an analytic modified power-law distribution, (2) scaled to the published densities from a direct simulation Monte Carlo (DSMC) simulation that comprehensively simulates the entire thermal and sputtered neutral atmosphere^3^ and (3) scaled to a solely sputtered source^20^.

We find that a neutral atmospheric H_2_ density profile $n\left(r\right)={n}_{0}\exp(-h/\lambda){r}^{-2}$ (ref. ^37^) is able to reproduce the observed PUI density profile. Using a ${\chi }^{2}$ fit, we find that the surface density *n*_0_ = 1.8 ± 0.05 × 10^5^ cm^−3^ and *λ* = 6,090 ± 890 km fit the JADE PUI observations. Both the DSMC full-atmosphere simulation and sputtered-only simulation, when scaled with the JADE data to their peak expected densities, underpredict the radial profile of observed H_2_^+^ PUIs. This finding is insensitive to a reasonable range of streamline model parameters (Methods). In addition to being the dominant ionizing process, electron impact ionization is found to be the overall dominant loss mechanism for H_2_ at Europa’s orbital distance within Jupiter’s magnetosphere. Using these neutral profiles with our derived electron impact ionization rates, we find the total atmospheric loss rate as PUIs (Methods) to be 0.16 ± 0.04 kg s^−1^ (4.8 ± 0.1 × 10^25^ s^−1^). Comparing with previous H_2_^+^ ion observations in Jupiter’s magnetosphere^29^, we infer 6–41% of Europa’s escaping H_2_ neutrals are directly lost from the atmosphere as H_2_^+^ PUIs (Methods). Much of the remaining atmospheric losses will be in the form of neutrals, which populate a neutral toroidal cloud co-orbiting with Europa^3,29,38–40^. This process should also be occurring to varying degrees at Ganymede^41^ and Callisto^42^. An even smaller fraction would leave the Jovian system as unbound energetic neutral atoms^38^.

In the dawn-side region, we estimate Juno to be connected to streamlines that probe to less than 250 km altitude (inset axis in Fig. 3). H_2_ neutrals with a thermal speed distribution driven by Europa’s 86–132 K surface temperatures^43^ would have scale heights of 270–415 km. Hence, we can assess from our advection analysis the total content of the neutral atmospheric H_2_ population within a single thermal scale height. The finding that a profile of $n\left(r\right)={n}_{0}\exp(-h/\lambda){r}^{-2}$ can fit the observed PUIs suggests the atmospheric neutral population is not thermalized. This is also supported by comparison with the full-atmosphere DSMC simulation from ref. ^3^, which is dominated by thermalized neutrals and is not consistent with the observations. Such a finding is contrary to the prevailing understanding before the Juno fly-by that H_2_ neutrals in the atmosphere would have all three of the following properties: (1) They predominantly leave the surface with a thermal speed distribution closely matching the local temperature of the surface^2^. (2) They have small scale heights ~270–415 km. (3) Their speed distribution is not further modified. Additionally, the comparison in Fig. 3 with a sputtered-only model^20^ shows the observed population is also not consistent with a completely sputter-driven population. The radial profile we find, which is steeper than *r*^−2^, indicates that there is a predominantly escaping neutral population, which would follow *r*^−2^, that is also being ionized and depleted to steepen the radial neutral profile, as discussed in the next section.

The vertical neutral atmospheric column density (Methods) along the radial direction from the centre of Europa is calculated to be 1.8 ± 0.1 × 10^13^ cm^−2^ for H_2_ from the inferred non-thermal neutral population. Before Juno’s fly-by, this value had not been observationally constrained^7^. The H_2_ column densities derived here are a factor of ~4 smaller than those estimated from ref. ^3^ of 7.7 × 10^13^ cm^−2^, comparable to the value from ref. ^21^ of 2.5 × 10^13^ cm^−2^ and an order of magnitude higher than the sputtered-only value from ref. ^20^ of 1.9 × 10^12^ cm^−2^.

Unlike for H_2_, for which the thermal neutral scale heights are comparable to Juno’s fly-by altitudes, the expected scale heights for O_2_ are tens of kilometres (refs. ^4,20^). Due to this, we do not derive the total loss rate of O_2_ directly from the O_2_^+^ observations as we have done for H_2_. PUIs from the denser thermal O_2_ atmosphere may be highly concentrated in the most central portion of the wake and Juno may not have directly observed PUIs from this portion of the atmosphere. Therefore, we do not derive properties of the O_2_ neutral atmosphere. However, the Juno fly-by still reveals important information about the evolution of neutral O_2_. Electron impact ionization rates of 1.9 × 10^−6^ s^−1^ have been previously used to calculate modelled O_2_ losses^15^. The electron impact ionization rates derived here of 4.9 × 10^−6^ s^−1^ upstream from 9:37 to 9:39 during the fly-by are a factor of ~3 larger, and those in the wake of 3.3–8.1 × 10^−6^ s^−1^ are a factor of 2–4 times larger (Methods and Extended Data Table 3). The rate is proportional to the 1,356 Å O i emission rate used to derive the neutral column densities, which implies that this rate, at least for the time of the Juno fly-by, is also a factor of 2–4 larger. Consequently, to be consistent with brightness values measured remotely, we expect the O_2_ atmosphere to be a factor of ~2–4 times less dense compared to estimates using pre-Juno electron impact dissociation rates. Hence, the Juno fly-by observations are consistent with a lower O_2_ loss rate, both in the O_2_ electron impact ionization rates and the H_2_ loss rates that are a tracer for total O_2_ production.

## Discussion and conclusions

**Fig. 4:**
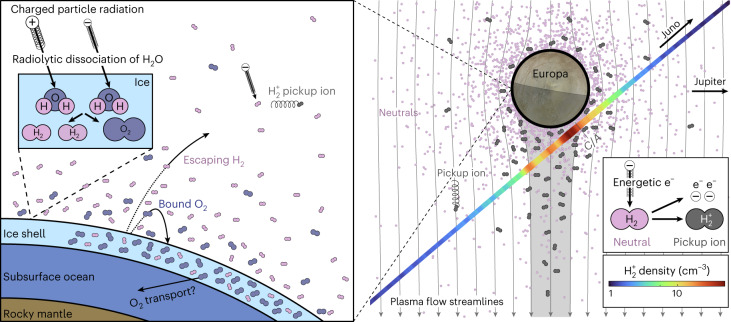
Overview of Juno’s Europa fly-by. Water ice on the surface of Europa is dissociated by radiolysis to form O_2_ and H_2_. These gases can migrate both inwards towards the subsurface ocean or escape the surface by thermal desorption or direct sputtering to form its atmosphere. The lighter H_2_ occupies a more extended region than heavier O_2_, which remains closer to the surface. A portion of the neutrals in the atmosphere are ionized and picked up by the magnetospheric plasma. Juno observes these PUIs, with the relative abundances driven by the various processes described here. The radiolysis dissociation inset was adapted from ref. ^2^. Particles shown are O_2_ (blue), H_2_ (pink) and H_2_^+^ (grey).

From a combination of Juno’s Europa fly-by and several transits through Europa’s orbit, we estimate Europa’s total neutral H_2_ loss rate to be 1.5 ± 0.8 kg s^−1^ (4.5 ± 2.4 × 10^26^ s^−1^). H_2_ is an effective tracer for the evolution of Europa’s surface ice. Observations of H_2_^+^ PUIs during Juno’s single fly-by of Europa and of H_2_^+^ PUIs throughout Jupiter’s magnetosphere taken over several years provide very similar loss rate estimates (Methods). Assuming that all oxygen produced by the radiolytic dissociation of H_2_O in the surface forms molecular O_2_ (ref. ^1^) and that the same process creating H_2_ produces O_2_ in a 2:1 ratio, we expect 12 ± 6 kg s^−1^ (2.2 ± 1.2 × 10^26^ s^−1^) of O_2_ to be produced in the top layer of Europa’s icy surface. This puts direct observational constraints on the pathways for O_2_ produced in the surface, such as the total loss rate of O_2_ from the atmosphere and O_2_ accessible to the subsurface ocean. Figure 4 and Table 1 summarize the surface processes and Juno observations made during its fly-by of Europa.

**Table 1:** Relevant parameters derived from Juno data

Parameter	Value
**Europa’s surface ice**	
H_2_ production	1.5 ± 0.8 kg s^−1^ (4.5 ± 2.4 × 10^26^ s^−1^)
O_2_ production	12 ± 6 kg s^−1^ (2.2 ± 1.2 × 10^26^ s^−1^)
H_2_O dissociation	13 ± 7 kg s^−1^ (4.5 ± 2.4 × 10^26^ s^−1^)
Surface erosion	1.5 ± 0.8 cm Myr^−1^ (95 ± 52 Myr m^−1^)
**Europa’s H**_**2**_ **atmosphere**	
H_2_ profile	$n\left(r\right)={n}_{0}{\mathrm{e}}^{-h/\lambda }{r}^{-2}$
H_2_ surface density	*n*_0_ = 1.8 ± 0.05 × 10^5^ cm^−3^
H_2_ ionization scale height	*λ* = 6,090 ± 890 km
H_2_ outflow speed	58 ± 34 m s^−1^
H_2_ loss	1.5 ± 0.8 kg s^−1^ (4.5 ± 2.4 × 10^26^ s^−1^)
Direct H_2_^+^ loss	0.16 ± 0.04 kg s^−1^ (4.8 ± 0.1 × 10^25^ s^−1^)

In the H_2_ profile relation, *h* is the altitude above the surface and *r* is the radial distance from Europa’s centre.

Due to radiolysis, the loss rates of H_2_ we derive require 13 ± 7 kg s^−1^ of water ice to be dissociated, which erodes Europa’s surface by 1.5 ± 0.8 cm Myr^−1^ (95 ± 52 Myr m^−1^). Galileo’s observations of impact ejecta from its Europa fly-bys^44^ were consistent with an impact ejecta mass loss of 0.2 kg s^−1^, corresponding to an erosion rate of 0.2 mm Myr^−1^, assuming that pure ice is ejected, which is more than an order of magnitude lower than that from the radiolysis-driven dissociation of surface ice calculated here. Additionally, as the top 30 cm of the surface is anticipated to be impact gardened over tens of millions of years (ref. ^45^), even with the modest H_2_O loss rates derived here, the radiolysis-driven erosion of the surface is comparable to, if not the dominant driver of, Europa’s surface erosion and modification. These updated constraints also affect the preservation of potential biosignatures in Europa’s near-surface ice layers^46^.

Historically, the neutral H_2_ atmosphere was understood to be dominated by a thermalized population with a speed distribution like that of the local surface temperature^2,4^. In contrast to expectations, we find the neutral H_2_ atmosphere is dominated by a non-thermal population with a radial dependence of $n\left(r\right)={n}_{0}\exp(-h/\lambda){r}^{-2}$, as has been employed for Io’s atmospheric escape^37^. Such a radial distribution would arise from an outflowing, escaping neutral population (${r}^{-2}$ dependence) that incurs losses ($\exp(-h/\lambda)$ dependence), which is directly observed for the H_2_ + e^−^ → H_2_^+^ + 2e^−^ pathway as PUIs. From this altitude profile (Fig. 3), we find the average neutral outflow speed is 58 ± 34 m s^−1^. We independently estimate the total neutral outflow loss (Methods), which nearly identically matches the values derived relying primarily on Europa-genic H_2_^+^ PUI observations far from Europa^29^. Therefore, the H_2_ neutral altitude profile and the total derived H_2_ loss rates are independently consistent, giving further confidence that the H_2_ population is non-thermal and has been heated after release from the surface by an additional mechanism. Although we cannot address the heating mechanism for such a population with this current analysis, it may be the result of atmospheric sputtering^15^, direct surface sputtering^20,21^, Joule heating^47^ or a combination of these effects. Joule heating is a favourable candidate, as such a process is most efficient when the interaction strength (Methods) $\bar{\alpha }=0.5$ (ref. ^48^), and we have found $\bar{\alpha }=0.55$ to represent the observations well.

The overall budget of 12 ± 6 kg s^−1^ total O_2_ produced in the surface is partitioned into atmospheric loss and potential sequestration into the surface ice. The loss of neutrals from the surface is often termed the ‘source rate’ in the literature, which is equal to the production rate if all neutrals eventually make their way to the surface or is less than the production rate if an appreciable fraction of neutrals are transported downward away from the surface. Before Juno’s transit of Europa, model-driven estimates for the total Europa-genic O_2_ source extended over two orders of magnitude^2,7,20,49^ from 5 to 1,100 kg s^−1^. Here, we constrain this value to less than 12 ± 6 kg s^−1^, as the production rate is an upper limit on the atmospheric source rate and is in the very lowest range of previous estimates. Previous modelling efforts provide context to the relative magnitude of oxygen production. A modelling study investigating the physics of O_2_ production and ejection from the surface found production rates of 8–26 kg s^−1^ to be consistent with O_2_ forming a thin layer near the surface, compared to 430–1,100 kg s^−1^ for a thick layer, for which the oxygen reservoirs exist deeper than the penetration depth of magnetospheric ions^7^. As the thin-layer hypothesis and corresponding modelled production rates are similar to the observational constraints found here, these results are consistent with the notion proposed by ref. ^7^ that oxygen could reside in a narrow layer near the surface. A separate modelling parameter study^16^ showed that with upstream densities of 100 cm^−3^ like those observed on the Juno fly-by, our production rates of 12 ± 6 kg s^−1^ are consistent with Europa having a small height for neutral O_2_ of approximately tens of kilometres.

With respect to potential transport downward and away from the surface, radiolytically produced O_2_ retained in Europa’s ice may work its way into the ocean as a possible source of metabolic energy for life^50^. Estimates of current O_2_ delivery from the oxygenated ice to the liquid ocean range from 0.3 to 200 kg s^−1^ (ref. ^51^) up to 300 kg s^−1^ (ref. ^52^). Unless Europa’s oxygen production was significantly higher in the past, the O_2_ production rates found here of less than the 18 kg s^−1^ available to be retained in Europa’s surface ice provide a narrower range to support habitability than previous model-driven estimates.

## Methods

### PUI energy

PUIs are injected at a velocity in the corotating frame of $\mathbf{v}_{\mathrm{PUI,cor}}=\mathbf{v}_{\mathrm{cor}}-\mathbf{v}_{\mathrm{orb}}$, where ${v}_{\mathrm{cor}}=\omega r\cos \theta$ is the corotational speed, *r* is the radial distance, ${v}_{\mathrm{orb}}=\sqrt{\mu /r}$ is the orbital speed, *ω* = 1.757 × 10^−4^ s^−1^ is Jupiter’s angular rotation frequency (period of 9.93 h), *μ* = 1.267 × 10^17^ m^3^ s^−1^ is Jupiter’s standard gravitational parameter and $\theta$ is the latitude. In a reference frame centred on Jupiter but not rotating with the planet, PUIs have a speed in the range from ${v}_{\mathrm{PUI,inj}}=\left|2\mathbf{v}_{\mathrm{cor}}-\mathbf{v}_{\mathrm{orb}}\right|$ to ${v}_{\mathrm{PUI,min}}={v}_{\mathrm{orb}}$. Juno’s relative motion plays a role in the detected PUI energies. Hence, the peak observed speed expected in the spacecraft frame for PUIs is ${v}_{\mathrm{PUI,Juno}}=\left|\mathbf{v}_{\mathrm{PUI,inj}}-\mathbf{v}_{\mathrm{Juno}}\right|=\left|\mathbf{v}_{\mathrm{Juno}}-2\mathbf{v}_{\mathrm{cor}}+\mathbf{v}_{\mathrm{orb}}\right|$, where $\mathbf{v}_{\mathrm{Juno}}$ is the velocity vector of the Juno spacecraft with respect to Jupiter’s centre in a non-rotating frame.

### Density determination from TOF data by mass range

Although JADE’s TOF product does not have directionality information, it does observe the full sky each ~30 s spin. We calculate partial numerical densities from the count rates as a function of energy over the JADE energy band-pass for each sample period of 2 s and apply a sliding average over a full 30 s spin. After all foregrounds and backgrounds are subtracted (see Supporting Information in ref. ^29^), we sum count rates over all TOFs corresponding to *M*/*q* between the mass ranges 1.5–2.5 for H_2_^+^ and 26–70 for O_2_^+^ and S^+^ to determine a total count rate *R*_obs_ as a function of energy. Count rates for H^+^ are derived from existing proton foreground removal methods used to isolate H_2_^+^. For all species-specific count rates, we subtract the average count rates per energy in the *M*/*q* range of 2.75 to 5.1 to remove the long TOF tail from O^+^ and S^++^ ions that are foreground to other mass ranges (Supporting Information in ref. ^29^).

JADE instantaneously observes an angular range of 270° extending from the anti-sunward spin axis, such that for each spacecraft rotation, it records counts from a total angular extent of 6π sr, double-counting half the sky. We must reduce *R*_obs_ by an appropriate factor to determine the ‘true’ average count rate *R* *=* *ηR*_obs_ corresponding to the 4π sr full sky. Due to the instrument mounting and orbit geometry, for each observation by JADE, the plasma incident on JADE is predominantly observed on the hemisphere where JADE double-counts incident populations, which also gives improved counting statistics. Following previous analyses^29^, we use *η* = 0.5.

We then convert count rate *R* into phase space density *f* using $f(v)=R/({G}_{\mathrm{eff}}^{v}\left(v\right){v}^{4})$, where ${G}_{\mathrm{eff}}^{v}={G}_{\mathrm{eff}}^{E}/2$ is the energy-dependent geometric factor^53^, with a factor of two between the energy geometric factor and velocity geometric factor^54^, and *v* is the measured energy per charge converted to speed for each given species mass. In turn, the number density derived from a one-dimensional phase space density is ${n}_{\mathrm{num}}=4\pi {\int }_{0}^{\infty }f\left(v\right){v}^{2}\,\mathrm{d}v$. For JADE data with count rates in discrete energy bins, the numerical partial number density is given by ${n}_{\mathrm{num}}=(4\pi/3)\sum_{i}\left({v}_{i,\max }^{3}-{v}_{i,\min }^{3}\right)f({v}_{i})$, where *i* indicates each energy bin that spans in velocity space from *v*_*i*,min_ to *v*_*i*,max_ and *v*_*i*,max_ = *v*_*i*+1,min_.

### Electron impact rates

The JADE electron observations during Juno’s Europa transit are taken with two 120° × ~5° field-of-view electron sensors (JADE-E), covering a total of 240° along the plane perpendicular to Juno’s spin axis. They can electrostatically deflect up to 35° towards the direction of the local magnetic field direction to capture field-aligned electrons. Electron intensities (cm^−2^ sr^−1^ s^−1^ keV^−1^) as a function of energy *E* and pitch angle *θ*, *I*(*E,θ*), are derived from count rates. The total reaction rate *γ* is given by

$$\gamma =\iint I(E,\theta )\sigma (E)\,\mathrm{d}E\,\mathrm{d}\varOmega =2\pi {\int }_{0}^{\pi }\sin\theta\,\mathrm{d}\theta {\int }_{{E}_{{\min }}}^{{E}_{{\max }}}I(E,\theta )\sigma(E)\,\mathrm{d}E,$$

where the differential solid angle is from assuming gyrotropy. For JADE-E’s energy range during the Europa fly-by, *E*_min_ = 30 eV and *E*_max_ = 40 keV. We estimate these reaction rates using the energy-dependent cross-sections *σ*(*E*) for each reaction and species (Extended Data Table 3).

Since JADE-E does not measure electrons below ~30 eV, it misses a small portion of the relevant ionizing electron population below this energy. We extend the intensities below JADE-E’s energy range by fitting kappa distributions to the electron intensity spectra (example given in Extended Data Fig. 2), following results from an empirical model that reproduced previous electron observations at Jupiter^55^. Integrating the above equation from *E*_min_ = 0 eV using the empirical model intensities below 30 eV, we find reaction rates that are 10–30% larger than those solely using JADE-E’s lower-energy limit of *E*_min_ = 30 eV.

### O_2_^+^ density determination

The JADE instrument cannot isolate species with the same mass per charge, hence the *M*/*q* = 32 data product contains fluxes from S^+^ and O_2_^+^ and may also contain false coincidences from O^+^ and S^++^ on the lower-energy end of the observed flux enhancements. We isolate and extract the signature of fresh O_2_^+^ PUIs using a data-driven method described below. Although modelling the specific instrument response to different species can be used to extract composition ratios^53^, we apply a strictly data-driven approach to estimate O_2_^+^ densities. Since the H_2_^+^ ions are unambiguously local Europa-genic PUIs, their energy spectra give a data-driven representation of a nominal PUI. We assume O_2_^+^ PUIs will have a similar distribution, scaled up by a factor of 16 in energy due to their mass ratio to H_2_^+^. Therefore, we use the shape of the H_2_^+^ PUI distribution to apply a mask to the O_2_^+^ data and derive densities from this mask.

We derive a mask from the H_2_^+^ spectrogram by finding contours in the H_2_^+^ flux that occur within a certain percent of the peak for each time step. Extended Data Fig. 1b shows this mask. The rates below those of 20%, 40% and 60% from the peak flux have been masked out. From this masked data, we calculate the H_2_^+^ density again, finding it to be lower than that derived for the entire distribution. We then calculate the correction factor that we would need to scale the mask-derived densities to reach the correct values, as shown in Extended Data Fig. 1d. We then apply the mask to the O_2_^+^ dataset, scaled up in energy by a factor of 16 (Extended Data Fig. 1a), calculate the density for the masked O_2_^+^ dataset and then apply the same correction factor. Finally, we subtract the derived density using this method upstream of Europa at 9:39 to remove the contribution from foreground magnetospheric ions not of Europa-genic origin. The dashed orange lines in Extended Data Fig. 1c show the range of O_2_^+^ densities we derived for values 20–60%.

As shown in Extended Data Fig. 1a,d, the higher-energy *M*/*q* = 32 population very nearly tracks the energy distribution expected based on H_2_^+^ PUIs. Therefore, we attribute the higher-energy ions at *M*/*q* = 32 to fresh O_2_^+^ PUIs from Europa’s atmosphere picked up in similar locations and conditions to H_2_^+^. The range of densities for O_2_^+^ found with this technique is shown in Fig. 1f.

### PUI advection model

The PUI density at any location is determined by the net pickup upstream along the streamline intersecting that point. We employ a simple streamline model originally developed for Io’s plasma interaction. The original formulation determined the velocity field as a function of the Peterson conductance ${\varSigma }_{1}$ and Alfvénic conductance ${\varSigma }_\mathrm{A}$ (Appendix A2 in ref. ^56^ and Section 2.1.2 in ref. ^48^). Here, we reformulate the velocity field to depend on two unknown parameters: (1) the interaction strength $\bar{\alpha }=\frac{{\varSigma }_{1}}{{\varSigma }_{1}+{2\varSigma }_\mathrm{A}}=\frac{\delta v}{{v}_{0}}$ and (2) ionospheric distance ${R}_\mathrm{I}$, where ${v}_{0}$ is the unperturbed flow speed and $\delta v$ is the maximum change of the total flow speed. The plasma flow velocity vector is then given by:

$$\mathbf{v}_{\mathrm{B}}={v}_{0}\left[\begin{array}{c}1-\bar{\alpha}\\ 0\\ 0\end{array}\right]\quad{\rm{for}}\quad{{x}}_\mathrm{B}^{2}+{{y}}_\mathrm{B}^{2}\le {R}_\mathrm{I}^{2},$$

$$\mathbf{v}_{\mathrm{B}}={v}_{0}\begin{bmatrix}1-\bar{\alpha}{R}_\mathrm{I}^{2}({{x}}_\mathrm{B}^{2}-{{y}}_\mathrm{B}^{2})/{({{x}}_\mathrm{B}^{2}+{{y}}_\mathrm{B}^{2})}^{2}\\ -\bar{\alpha}{R}_\mathrm{I}^{2}\,2{x}_\mathrm{B}{y}_\mathrm{B}/{({{x}}_\mathrm{B}^{2}+{{y}}_\mathrm{B}^{2})}^{2}\\ 0\end{bmatrix}\quad{\rm{for}}\quad{x}_\mathrm{B}^{2}+{{y}}_\mathrm{B}^{2}> {R}_\mathrm{I}^{2},$$

where $\mathbf{v}_{\mathrm{B}}$ is in a coordinate system with −*z*_B_ aligned with the magnetic field axis (at Europa, the magnetic field is predominantly in the −*z*_EPhiO_ direction), +*x*_B_ is aligned with the flow direction (as in *x*_EPhiO_) and +*y*_B_ completes the right-handed system, as shown in Extended Data Fig. 5. The velocity in the EPhiO coordinate system requires a single rotation about the +*x*_B_ axis by angle $\varphi$ with a rotation matrix, such that $\mathbf{v}_\mathrm{EPhiO}=M\mathbf{v}_{\mathrm{B}}$, where

$$M=\left[\begin{array}{ccc}1 & 0 & 0\\ 0 & \cos \varphi & \sin \varphi \\ 0 & -\sin \varphi & \cos \varphi \end{array}\right].$$

During Juno’s fly-by, that angle is approximately $\varphi =12^\circ$ using the JRM09 internal field model^33^ and current sheet model^34^. However, the resulting velocity field values are not very sensitive to changes in this angle of ~5–10°. Note that this streamline model neglects the Hall effect, which has a minor effect on the ion flow in Europa’s ionosphere^15,57^.

Extended Data Fig. 6 compares this two-parameter model with the in situ speeds measured by JADE for $\bar{\alpha }=(0.4,0.55,0.7)$ and ${H}_\mathrm{I}={R}_\mathrm{I}-{R}_{\mathrm{Eur}}=(30\,{\rm{km}},100\,{\rm{km}},300\,{\rm{km}})$. Three model curves are shown in each time series, corresponding to rigid corotation at 104 km s^−1^ with respect to Europa along with sub-corotation speeds of 100 and 95 km s^−1^ as reasonable possibilities^27^. Overall, we find this model successfully replicates the flow speeds and trends observed by JADE. Specifically, the model predicts a depletion in speed as Juno transits near the centre of the wake, followed by a speed enhancement as Juno encounters the sub-Jovian flank where streamlines are compressed (leading to increased plasma speed) to divert around Europa. We choose the value of ${H}_\mathrm{I}=30$ km here in our analysis as the dominantly O_2_ atmosphere is understood to have a scale height of tens of kilometres^4,20^, but note that the results are not very sensitive to this choice, as discussed below. For the interaction strength, we find that lower values of $\bar{\alpha }=0.4$ underpredict the observed speed variations, whereas $\bar{\alpha }=0.7$ overpredicts them. Hence, we use an intermediate value of $\bar{\alpha }=0.55$ for our analysis, but as discussed below, the results are relatively insensitive to the specific choices of $\bar{\alpha }$ and ${H}_\mathrm{I}$.

To determine the PUI density for a specific set of plasma flow and neutral atmosphere conditions, we solve the continuity equation for an advecting plasma with a source, $\frac{\partial n}{\partial t}+\nabla \cdot\left(n\mathbf{v}\right)=P$, where $n$ is the PUI density, $\mathbf{v}$ is the plasma flow velocity from the model described above and *P* is the PUI injection source term. Let $P=\gamma {n}_\mathrm{a}(\mathbf{r})$, where $\mathbf{r}$ is the radial vector from the centre of Europa, $\gamma$ is the ionization rate and ${n}_\mathrm{a}(\mathbf{r})$ is the atmospheric neutral density assuming a radially symmetric profile. Assuming the flow is in one dimension *s* along the streamline and that the density profile is not explicitly dependent on time, then we can solve $v\frac{\partial n}{\partial s}=\gamma {n}_\mathrm{a}(\mathbf{r})$ for the PUI density at Juno’s location using finite differences along a streamline, such that ${n}_{i+1}={n}_{i}+\frac{\gamma \Delta s{n}_\mathrm{a}(\mathbf{r}_{i})}{{v}_{i}}$. Assuming the neutral density ${n}_{0}=0$ far upstream and using a constant step size $\Delta s$, the local PUI density at Juno’s location for a given species is then given by:

$$n=\gamma \Delta s\sum _{i}\frac{{n}_\mathrm{a}\left(\mathbf{r}_{i}\right)}{{v}_{i}}.$$

We use a small step size of $\Delta s=0.05\,R_\mathrm{E}$, such that the results are not sensitive to this choice. The atmospheric profile considered is ${n}_\mathrm{a}(r)={n}_\mathrm{a0}\exp(-h/H)r^{-2}$, where *h* is the altitude above the surface accounting for Europa’s oblateness (it has an equatorial radius of 1,560.8 km and a flatness coefficient of 1.98 × 10^−3^), *H* is the atmospheric scale height, and *r* is the radial distance from Europa’s centre. Two additional published atmospheric profiles are also included for comparison^3,20^. We apply this advection model to the period after Juno exits Europa’s geometric wake starting at 2022-272 09:36:29. The fit shown in Fig. 3 is derived using $\bar{\alpha }=0.55$ and ${H}_\mathrm{I}=30$ km. However, we tested the sensitivity of these results to changes in the two components in the model in the range discussed above. In this sensitivity investigation, we found the overall interpretation and extraction of atmospheric profile was highly insensitive to the choice of either parameter, with the exception of ${H}_\mathrm{I}=300$ km. For large scale heights like 300 km, the flow would be appreciably slowed near Juno’s close approach leading to a substantial perturbation in PUI densities that JADE did not observe. Hence, overall the derived results are robust to changes in the plasma interaction within a reasonable parameter space.

### Atmospheric profile calculations

The column density along the radial direction for an atmospheric density profile ${n}_\mathrm{a}(r)$ is $\int {n}_\mathrm{a}(r)\,\mathrm{d}r$. For an exponential altitude with scale height *H* = *kT*/*mg*, the thermal energy per kilometre scale height is 4.4 × 10^−4^ eV km^−1^ or 5.1 K km^−1^ for Europa’s surface gravity of *g* = 1.315 m s^−2^ and Boltzmann’s constant *k* = 1.38 × 10^−23^ m^2^ s^−2^ K^−1^ = 8.62 × 10^−5^ eV K^−1^.

### Determination of H_2_ and O_2_ production rates

Since H_2_ is more readily released from Europa’s surface and gravitational well, the total H_2_ production rate allows us to directly estimate the total O_2_ production rate within Europa’s icy surface. The O_2_ mass production rate is assumed to be 8 times the H_2_ production rate from the stoichiometric ratio of hydrogen and oxygen in H_2_O. We determine the total H_2_ production rate in Europa’s icy surface using Juno’s Europa fly-by data, Juno measurements of the electron characteristics in the vicinity of Europa’s orbit and Juno observations of H_2_^+^ produced from Europa’s neutral H_2_ loss.

The previous estimate of total H_2_ loss rate from Europa^29^ did not account for PUIs directly lost from Europa and relied on Voyager/Galileo electron characteristics to determine loss rates in the vicinity of Europa’s orbit. Previous reaction rate estimates^40^ found that 86–91% of all reaction pathways for neutral H_2_ were due to electron impacts. Therefore, we focus on updating these reactions with Juno measurements, as they are the overwhelmingly dominant reaction pathways. The recent fly-by along with Juno measurements in the vicinity of Europa’s orbit allow for a direct determination of the electron impact ionization rates in both environments. We use Juno/JADE observations to improve the electron impact rate estimates and separately estimate the total losses from Europa’s atmosphere versus within the neutral toroidal cloud.

First, we determine the electron distribution function for all times Juno was within 9 to 10 *R*_J_ and within 2 *R*_J_ from the magnetic equator (Extended Data Table 2). An example set of spectra is shown in Extended Data Fig. 2. We then fit the electron intensity profile as discussed in the ‘Electron impact rates’ in Methods and determine electron impact ionization rates for all time periods. We bin these rates by magnetic latitude (Extended Data Fig. 3) and perform a weighted average for each latitude bin by the time Europa spends in each magnetic latitude. In this way, we can determine the average electron conditions experienced by neutrals in Europa’s orbit (Extended Data Fig. 4 and Extended Data Table 3) using all relevant reaction cross sections^40,58–62^.

To directly estimate the total PUI loss rate from Europa’s neutral atmosphere, JADE must transit streamlines that sample the majority of the neutral H_2_ column density. As Juno transits streamlines down to 200–300 km within a single thermal scale height, it will sample nearly the entire neutral atmosphere. A single population with a radial dependence of $n(r)={n}_{0}\exp(-h/\lambda){r}^{-2}$ (ref. ^37^) is capable of completely fitting the observed PUIs. By comparison, the comprehensive neutral DSMC model^3^ of the atmosphere dominated by thermal neutrals underpredicts the total PUI content. Hence, we find that a thermally dominated population of neutral H_2_ is not the dominant producer of the observed H_2_^+^ PUIs. Given this, we conclude that the observed PUIs represent the dominant losses, enabling us to estimate the total H_2_ loss.

Given the dawn-side observation we use to infer global atmospheric characteristics, we investigate two cases to bound this estimate: (1) a radially symmetric atmosphere and (2) a case where the neutral H_2_ dawn/dusk asymmetry is a factor of 2, which is the upper limit of the dawn/dusk column densities observed in O_2_ by the Hubble Space Telescope^4,11^. We use the time range of 9:37 to 9:39 as representative upstream conditions, such that the average upstream electron impact ionization reaction rate is 3.4 × 10^−6^ s^−1^. We further assume that half of the H_2_ neutrals in the downstream hemisphere experience a median reaction rate of 5.2 × 10^−6^ s^−1^ from the wake (Extended Data Fig. 4). Combining these two rates, we derive a local-time-averaged reaction rate of 4.3 × 10^−6^ s^−1^. Using our derived electron impact ionization rates to integrate over Europa’s entire atmosphere in these cases, the resulting estimate is 0.14 ± 0.03 kg s^−1^ of direct H_2_^+^ PUI loss.

From our updated electron reaction rates, along with existing non-electron-related rates^40^, we find that electron impact ionization, H_2_ + e^−^ → H_2_^+^ + 2e^−^, leads to 41–58% of total H_2_ losses in Europa’s orbit away from the moon, whereas 54–58% of H_2_ losses occur in the immediate vicinity of Europa. A production rate of 0.7 ± 0.3 kg s^−1^ of charged H_2_^+^ was derived from H_2_^+^ observations throughout Jupiter’s magnetosphere^29^. We now discriminate between H_2_^+^ directly lost from Europa’s atmosphere exposed to higher electron impact ionization rates with those picked up from Europa’s neutral H_2_ toroidal cloud. From the Europa fly-by, we estimate 0.16 ± 0.04 kg s^−1^ of these PUIs are directly picked up in the immediate vicinity of Europa from its atmospheric neutral H_2_. This leaves 0.54 ± 0.34 kg s^−1^ of ions to be produced from Europa’s neutral toroidal cloud. Using the relative fraction of impact ionization found here, 0.29 ± 0.09 kg s^−1^ of neutral H_2_ is lost directly from the Europa’s atmosphere due to H_2_ reactions, whereas the majority of neutral loss is from the torus and estimated to be 1.20 ± 0.72 kg s^−1^. The total estimated loss rate for H_2_ is then 1.5 ± 0.8 kg s^−1^. Using stoichiometric ratios for water, the total O_2_ production rate is then 12 ± 6 kg s^−1^.

Independently, the altitude profile of $n(r)={n}_{0}\exp(-h/\lambda){r}^{-2}$ can be used to estimate the total neutral H_2_ outflow. The length scale over which neutral losses occur can be interpreted to be $\lambda =w/L$, where $w$ is the average neutral outflow speed and $L$ is the total reaction rate for H_2_. For the total reaction rate, we follow a similar analysis as above. Averaging the upstream and wake electron impact ionization rates gives an average value that is ~80% of the wake value. Therefore, we sum all reaction rates derived for the H_2_ in the third column of Extended Data Table 3 for the wake and multiply by 80% to determine the average value of these electron-driven rates to be 3.8–11 × 10^−6^ s^−1^. We additionally estimate from the second column in this table that non-electron-driven rates can contribute an additional 20%, so we estimate a total H_2_ reaction rate of 4.6–13 × 10^−6^ s^−1^. The outflow speed is then $w$ = 58 ± 34 m s^−1^. To estimate the total loss rate with this outflow approximation, we similarly assume that the surface density is either azimuthally symmetric with *n*_0_ = 1.8 ± 0.05 × 10^5^ cm^−3^ or has a dawn/dusk asymmetry of 2 with an average surface density of *n*_0_ = 2.7 ± 0.08 × 10^5^ cm^−3^. The total neutral loss rate estimate is then $4\uppi {R}_{E}^{2}{n}_{0}w$ = 1.5 ± 1.1 kg s^−1^, which is remarkably similar to our higher-fidelity estimate of 1.5 ± 0.8 kg s^−1^ above, which was estimated in a completely different way using years of Juno observations of Europa-genic H_2_^+^ PUIs throughout the magnetosphere.

### Source data

Source Data for Fig. 1. Source data.

Source Data for Fig. 2. Source data.

Source Data for Fig. 3. Source data.

## Figures and Tables

**Extended Data Table 1:**
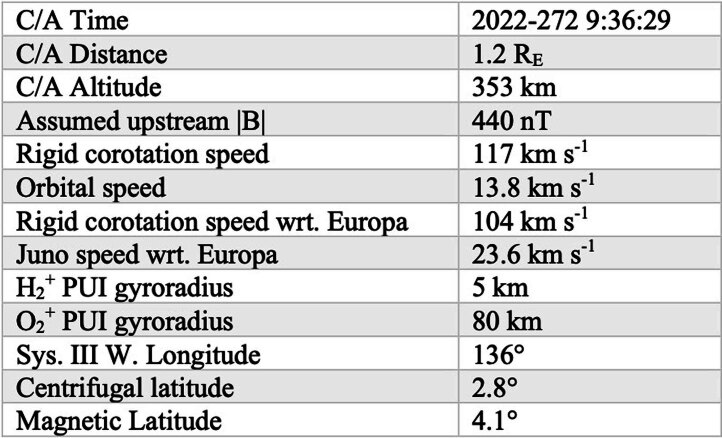
Relevant orbital and plasma parameters at close approach

All parameters calculated at Juno’s close approach on 2022-272 9:36:29.

**Extended Data Table 2:**
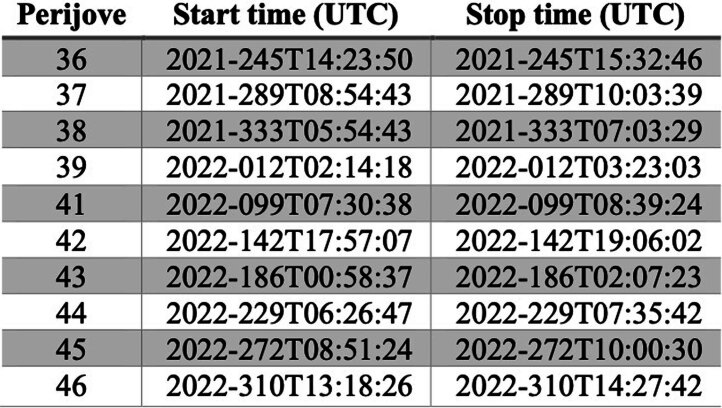
Times used for the electron rates derivations

Time ranges are when Juno was within 9-10 R_J_ radial distance from Jupiter and within 2 R_J_ vertical distance from the magnetic equator.

**Extended Data Table 3:**
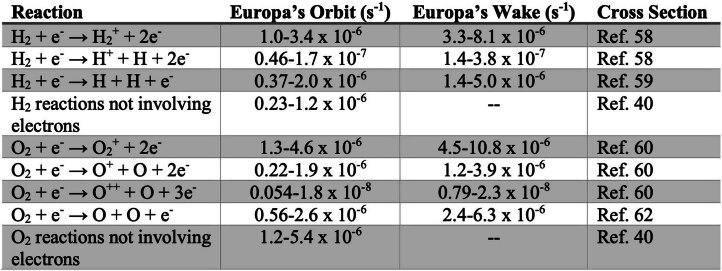
Electron reaction rates determined by Juno/JADE observations

Rates calculated both in the vicinity of Europa’s orbit and within Europa’s wake during Juno’s Europa flyby.

## Data Availability

The JNO‐J/SW‐JAD‐3‐CALIBRATED‐V1.0 data presented in this manuscript, 10.1007/s11214-013-9990-9, can be obtained from the Planetary Data System (PDS) at https://pds-ppi.igpp.ucla.edu/mission/JUNO/JNO/JAD. Source data are provided with this paper.

## Extended data

Extended data are available for this paper at 10.1038/s41550-024-02206-x.
